# HIV prevalence among female sex workers in Guigang City, Guangxi, China: an 8-year consecutive cross-sectional study

**DOI:** 10.1186/s12889-018-5380-2

**Published:** 2018-04-04

**Authors:** Jingzhen Lai, Chunwei Qin, Eric J. Nehl, Junjun Jiang, Yunxuan Huang, Bingyu Liang, Yuexiang Xu, Jiegang Huang, Zhiliang Xu, Chuanyi Ning, Yanyan Liao, Ning Zang, Wudi Wei, Fengxiang Qin, Jun Yu, Li Ye, Xionglin Qin, Hao Liang

**Affiliations:** 1 Guangxi Key Laboratory of AIDS Prevention and Treatment & Guangxi Universities Key Laboratory of Prevention and Control of Highly Prevalent Disease, School of Public Health, Guangxi Medical University, No. 22 Shuangyong Road, Nanning, 530021 Guangxi China; 2 Guigang Center for Disease Control and Prevention, No.7 Jianshexi Road, Guigang, 537000 Guangxi China; 3 Rollins School of Public Health, Emory University, 1518 Clifton Road. NE, Atlanta, GA 30322 USA; 4 Guangxi Collaborative Innovation Center for Biomedicine, Life Sciences Institute, Guangxi Medical University, No.22 Shuangyong Road, Nanning, 530021 Guangxi China

**Keywords:** HIV, AIDS, Prevalence, FSW, Guigang city, China

## Abstract

**Background:**

Female sex workers (FSW) are a population that are at high risk for HIV infection, and their HIV/AIDS knowledge levels and sexual behaviors are of concern. This study describes changes in HIV prevalence and factors associated among female sex workers in Guigang City, Guangxi, one of the highest HIV prevalence areas in China.

**Methods:**

Data were derived from an annual cross-sectional venue-based survey, 2008 to 2015, in the form of sentinel surveillance. The participants were recruited using cluster sampling. FSW aged 16 years and above who completed a questionnaire and HIV testing. Both descriptive and multi-level analyses were used to explore factors associated with changes in HIV prevalence.

**Results:**

Seven thousand four hundred ninety-six FSW were recruited in this study. HIV prevalence among FSW in Guigang City fell into two periods, one with an increasing trend (2008–2011) and one with a decline (2012–2015). Differences between these time periods included age, relationship status, HIV knowledge, consistent condom use, lifetime illicit drug use, history of sexually transmitted infection in the past year, HIV testing, receipt of a condom distribution and education program or HIV counseling and testing, and peer education services.

**Conclusions:**

Since 2012, a reduction in HIV prevalence among FSW in Guigang City has been observed. The decline of HIV prevalence was associated with coinciding changes in demographic characteristics of FSW, improvement of HIV knowledge and safer sexual behaviors, and a program that promotes condom use, HIV counseling & testing, and peer education.

## Background

The Chinese governmental response to the HIV/AIDS epidemic has prioritized interventions to control the epidemic and implemented routine HIV testing in populations at high risk of infection such as injection drug users, sex workers, men who have sex with men, and plasma donors [1], but the absence of long-term data about vulnerable populations in remote areas remain a gap in the literature. In fact, HIV/AIDS has had consistent growth in several areas of Chinese society; with those living in southwest China, female sex workers (FSW), men who have sex with men, and injection drug users being at particular risk [2, 3]. Meanwhile, prevalence in the Chinese general population remains low, pointing to the need for continued targeted efforts to control the epidemic. Recognition of the problem among these vulnerable groups is encouraging and systematic reviews have shown positive impacts of targeted behavioral interventions [4, 5]. However, challenges of culture, public health response, HIV stigma, and discrimination have continued to hamper successful response to these persistent health disparities [3].

FSW are a population at high risk for HIV acquisition worldwide [6]. A study of nearly 100,000 FSW across 50 countries found the overall HIV prevalence was 11.8%, with a pooled odds ratio for HIV infection of 13.5 when compared with women of reproductive age [7]. In China, a similar disparity exists, with the proportion of HIV infection among FSW being much higher than the general population [2]. HIV prevalence among FSW has approached or surpassed 1% in five Chinese provinces including Guangxi [8–11]. The HIV prevalence of FSW in low-grade venues is even higher than that of overall FSW, reaching 2.2% in Guangxi in 2011. Sex work has a long history in China, and although illegal, is widely practiced throughout the country [12]. Recent research indicates low rates of condom use, low HIV knowledge, multiple sexual partners, sexual violence, and high rates of other sexually transmitted infections (STI) among FSW in China [13–15]. HIV infection is also spread to the general population through sexual behaviors of FSW and their clients with their steady sexual partners, leading to “bridging” of HIV transmission [16, 17].

Guangxi, a province located in the southwest of China, is one of the highest-prevalence areas of HIV infection in China [18–20]. Guigang City, a prefecture-level city (comprised Guigang City, Guiping City and Pingnan County) with the third largest population in the province (5.43 million residents in 2014) [8], is the largest inland port city in South China and is situated between the large metropolitan areas of Nanning and Guangzhou. With its position as a trading and travel route between these two large cities, the market for commercial sex has led to a sizable population of FSW in Guigang City. Unsurprisingly, Guigang City has become one of the cities with more than total of 5000 new HIV infected cases reported during 2008–2013 [9]. However, the HIV prevalence among the key population of FSW in Guigang City remains unknown.

Since the State Council AIDS Working Committee Office of People’s Republic of China was established in 2004, national HIV/AIDS policy has supported HIV/AIDS prevention and control [1, 10]. Furthermore, Guangxi province has implemented its own HIV/ AIDS prevention and treatment program since 2010, aiming at reducing new infections, decreasing the fatality rate, and improving the quality of life for HIV/AIDS patients. High-risk populations are offered free condoms and education, HIV counseling & testing, community methadone maintenance treatment, clean needle and syringe exchanges, and peer education. Most specific to FSW, efforts directed at condom distribution, education, and HIV counseling and testing aim to promote consistent condom use and uptake of HIV testing. Additionally, peer education has been used as an approach to promote change among high-risk populations across the province. Although these programs are all available in Guangxi province [15] and has been implemented by public health workers in Guigang City, at this time it is unclear if they have been effective.

Focusing more intensely on smaller areas of China and among vulnerable groups such as FSW where HIV risk is still high is an important area of study that may shed light on high risk in an underserved group. Since there is very little information regarding the issue, the primary purpose of this study was to understand the prevalence of HIV infection among FSW in Guigang City based on a long-term annual cross-sectional survey. A secondary purpose was to explore demographic, sexual risk behaviors, and psychosocial correlates that either encourage or deter the behaviors that put individuals within this community at risk for increased STIs and HIV/AIDS over time. It is our hope that this research will further the literature which aims to understand HIV/AIDS among Chinese FSW, as well as act as a preliminary evaluation of local efforts into HIV/AIDS prevention and control. Information gathered could help guide further research and facilitate prevention efforts that are more appropriate and effective for FSW in other cities in Guangxi province, China as a whole, and provide direction for HIV prevention across Southeast Asia.

## Methods

### Study setting

Data for the current study were derived from sentinel surveillance implemented collected annually (2008 to 2015) from April to June with the use of a consecutive cross-sectional survey and objective HIV testing. Data were collected across three jurisdictions of Guigang City (including Guigang City, Guiping City and Pingnan County). A venue-based sampling frame was generated based on a map of commercial sex establishments maintained by the local Center for Disease Control and Prevention (CDC). The venues were sampled randomly from within this frame. Then FSW working in these venues were sampled by cluster sampling.

### Study population

To be eligible for the study, participants must have: (1) self-identified as a female; (2) been aged 16 and above; (3) been able to give verbal and written (in Mandarin) consent; and (4) self-reported receiving payment for sex in the past 6 months. Participants were verbally informed of the nature and purpose of the study, survey procedures, the sensitive nature of the questions, confidentiality parameters, and payment for participation. After informed consent forms were signed, trained interviewers aided participants in the completion of surveys that included questions from various domains, including socio-demographic characteristics, sexual history and recent sexual activity, lifetime illicit drug use, and HIV knowledge. Participants were compensated 50 Chinese Yuan at completion of the study for their time (typically one to two hours). Up to 400 participants were recruited in each of the three sentinel sites every year. If the number of the participants at any site was less than 250 per year, the sampling period was extended 4 weeks at most. This study was reviewed and approved by the Human Research Ethics Committee of Guangxi Medical University (Ethical review No. 2013–130).

### Measures

#### Socio-demographic characteristics

Participants were asked to report their date of birth, present legal residency or “hu-kuo” (Guangxi, or outside Guangxi), ethnicity (Han [the dominant ethnic group], Zhuang, or other), and education level. They were also asked to indicate their current relationship status (single or with a regular sexual partner they do not live with, married, cohabiting, or divorced or widowed).

#### HIV-related knowledge

HIV–related knowledge scale was measured using a scale developed by the Chinese National Center for AIDS/STD Control and Prevention [11]. Participants were asked eight questions relating to HIV risk practices. Response options were a dichotomous 0 = No, 1 = Yes, which were then added to create sum score (0–8) for each participant. Participants who gave six or more correct answers were coded as having sufficient knowledge of HIV/AIDS risk factors [11].

#### HIV-related risk behaviors

HIV-related risk behavior measures included: (1) the level of establishment in which the FSW worked, (2) consistent condom use in the past month, (3) lifetime illicit drug use, (4) history of a STI in the past year, and (5) lifetime HIV testing history. A combination of two items was used to measure the level of establishment in which participants worked. Participants were asked to identify (1) the mean income of FSW per transaction and (2) the type of venue they worked in. Those who reported working in establishments where the average cost was less than 100 Chinese Yuan earned per transaction and were located in a hostel, hair salon, or the street were identified as working in a low level venue (level 1); 100 to 200 Yuan earned per transaction in a hotel, footbath salon, or bar were identified as working in a middle level venue (level 2); and more than 200 Yuan earned per transaction with the venue being a bath house, night club or dancing hall were identified as working in a high level venue (level 3). Consistent condom use in the past month was assessed by asking participants the frequency with which they used condoms when having sex with their clients in the past month. Responses to this item used a three-point ordinal range, including “never used condoms during sex,” “used condoms during sex sometimes,” or “used condoms every time during sex.” Responses were coded into a single dichotomous measure of consistent condom use indicating those who always used condoms versus those who never or sometimes used condoms. Lifetime use of any illicit drugs was assessed by asking participants to report their lifetime use of drugs Response options were a 0 = No, 1 = Yes, and Refuse to Answer. Previous HIV/STI testing was assessed using two items. Participants were asked about their lifetime HIV testing and STI testing in the past year. Responses were coded as dichotomous (0 = No, 1 = Yes) and were analyzed separately. Last, participation in two governmental programs (1) condom distribution and education / HIV counseling & testing, and (2) peer education was assessed by two (0 = No, 1 = Yes) questions.

### HIV testing

Blood samples (3 mL venous blood) were collected by qualified nurses at the venue, and were tested in the laboratory department of Guigang CDC. Specimens were screened for HIV antibodies by enzyme-linked immunosorbent assay (ELISA; Beijing Wantai Biological Pharmacy Enterprise Co., Beijing, China). If the result of ELISA test was positive, participants were contacted by phone to come to the local CDC for confirmatory ELISA test (ELISA, Bio-Rad Laboratories Inc., Hercules, California, USA) and HIV-1 western blot tests (Diagnostics HIV Blot 2.2, MP Biomedicals Asia Pacific Pte Ltd., Singapore).

### Data analysis

The primary purpose of this study was to examine possible changes in HIV prevalence over the study period. First, all data were entered into a database by two different researchers and matched using EpiData 3.1 (The EpiData Association, Odense, Denmark). Second, descriptive statistics were used to characterize personal socio-demographics, HIV-related knowledge, HIV-related risk behaviors, and HIV testing results. Objective HIV testing data and prevalence were calculated using Microsoft Excel (Microsoft Corporation, Redmond, USA). Trend Chi-square tests were then used to examine changes in HIV prevalence across years and across the three sentinel sites using SPSS Statistics 16.0 (SPSS Inc., Chicago, IL, USA). Based upon these analyses which showed a significant change in the trend line in the period between 2008 and 2011 versus 2012–2015 (results described below) additional analyses with these time periods dichotomized were undertaken to explore factors that may influence why these changes in HIV prevalence occurred.

The secondary purpose of the study was to explore factors associated with HIV risk among FSW. Preliminary analyses were Chi-square tests to compare demographic characteristics, risk behaviors and participation in governmental programs among FSW between the 2008–2011 and 2012–2015 time periods. Multi-level fixed models were used to determine the associations between the risk factors and how they may have changed across the 2008–2011 and 2012–2015 time periods. All tests were two-sided, and *P* value equal or less than 0.05 was considered statistically significant.

## Results

### HIV prevalence among FSW

**Fig. 1:**
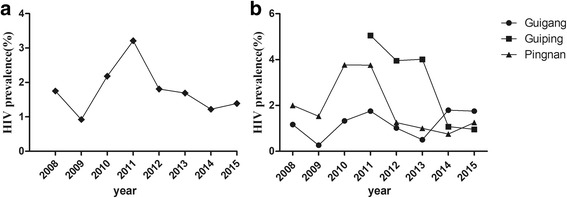
**a** The prevalence of HIV infection among FSW in Guigang City during 2008–2015, and **b** the HIV prevalence of FSWs in three jurisdictions in Guigang City during 2008–2015

A total of 73 FSW refused participation, 7496 FSW remained for analyses. The HIV prevalence, during 2008 to 2015, was 1.75% (10 / 571), 0.92% (7 / 764), 2.18% (20 / 916), 3.21% (32 / 997), 1.81% (19 / 1047), 1.69% (19 / 1123), 1.22% (13 / 1069) and 1.39% (14 / 1009), respectively. As shown in Fig. 1, between 2008 and 2009 there was a statistically significant decrease (Linear trend χ^2^_2008–2011_ = 7.496, *P* = 0.006) and from 2009 to 2011 there was a statistically significant increase in HIV prevalence, (Linear trend χ^2^_2009–2011_ = 10.514, *P* = 0.001). However, there were statistically significant decreases in HIV prevalence between 2011 to 2015 (Linear trend χ^2^ = 9.975, *P* = 0.002). Overall, HIV prevalence among FSW in Guigang City could be divided into two periods, (2008–2011) with an increasing trend and (2012–2015) with a declining trend. Despite of Guiping City started sentinel surveillance in 2011, HIV prevalence across the three sentinel sites were examined in order to understand if these trends varied by jurisdiction (Fig. 1a). Across, sites, the trends in Guigang City, Guiping City and Pingnan County were similar to the overall trend, indicating no geographic differences (Fig. 1b).

### Demographic characteristics of FSW

As can be seen in Table 1(see Table 1), from 2008 to 2011 most FSW (58.6%) ranged from 16 to 30 years old, whereas from 2012 to 2015 most FSW (62.9%) were between 31 to 50 years old. Over both time periods, most FSW were married (57.2% 2008–2011 and 73.4% 2012–2015). Hukuo, the registered residence assigned to all Chinese nationals, was approximately equally split between Guangxi province and outside Guangxi. Most FSW were Han ethnicity, and educational attainment was very low, with very few having attended high school. Chi-square analyses found statistically significant differences in age (*p* < .001), relationship status (*p* < .001), hukuo (*p* < .001), and education level (*p* < .001) between FSW recruited 2008–2011 and 2012–2015.

**Table 1:** Demographic characteristics of FSW in Period 2008–2011 and Period 2012–2015

Variables	Period 2008–2011	Period 2012–2015	*χ* ^*2*^	*P*
Age			4.46	< 0.001
16–30	1904 (58.6)	1464 (34.5)		
31–50	1318 (40.6)	2670 (62.9)		
> 50	26 (0.8)	114 (2.7)		
Relationship status			4.28	< 0.001
Single or regular sexual partner (not living together)	1184 (36.5)	717 (16.9)		
Married	1859 (57.2)	3120 (73.4)		
Cohabiting	127 (3.9)	124 (2.9)		
Divorced or widowed	78 (2.4)	287 (6.8)		
Hukou (Registered residence)			15.01	< 0.001
Guangxi	1820 (56.0)	2189 (51.5)		
Outside Guangxi	1428 (44.0)	2059 (48.5)		
Ethnicity			1.11	0.574
Han	2581 (79.5)	3361 (79.1)		
Zhuang	386 (11.9)	491 (11.6)		
Other	281 (8.7)	396 (9.3)		
Education level			1.36	< 0.001
Illiteracy	234 (7.2)	356 (8.4)		
Primary school	1190 (36.6)	1995 (47.0)		
Middle school	1476 (45.4)	1671 (39.3)		
High school	317 (9.8)	204 (4.8)		
College and above	31 (1.0)	22 (0.5)		
Total	3248	4248		

### Risk behaviors related to HIV infection of FSW.

As can be seen in Table 2 (see Table 2), 72.0% of FSW recruited between 2008 and 2011 gave at least six correct answers to HIV-related knowledge questions, compared to 96.6% of those recruited between 2012 and 2015 (*p* < .01). Between 2008 and 2011, few FSW (7.3%, 238 / 3248) had ever had a previous HIV test. However, 35.9% of those recruited 2012–2015 reported that lifetime HIV testing (*p* < .01). In addition, the level of establishment FSW worked (*p* < .01), consistent condom use in the past month (*p* < .01), and history of STI of participants in the past year (*p* < .01) differed significantly for those recruited 2008–2011 versus 2012–2015.

**Table 2:** Risk behaviors of FSW in Period 2008–2011 and Period 2012–2015

Variables	Period 2008–2011	Period 2012–2015	*χ* ^*2*^	*P*
HIV-related knowledge			9.18	< 0.01
Aware	2340 (72.0)	4103 (96.6)		
Unaware	908 (28.0)	145 (3.4)		
Level of establishment			1.61	< 0.01
Low (Level 1)	1797 (55.3)	2130 (50.1)		
Middle (Level 2)	724 (22.3)	1478 (34.8)		
High (Level 3)	727 (22.4)	640 (15.1)		
Consistent condom use in the last month			5.15	< 0.01
Yes	1358 (41.8)	2890 (68.0)		
No	1890 (58.2)	1358 (32.0)		
Lifetime illicit drug use			33.58	< 0.01
Yes	86 (2.6)	39 (0.9)		
No	3162 (97.4)	4209 (99.1)		
STI diagnosis in the past year			46.61	< 0.01
Yes	336 (10.3)	257 (6.0)		
No	2912 (89.7)	3991 (94.0)		
HIV tests previously			8.37	< 0.01
Yes	238 (7.3)	1527 (35.9)		
No	3010 (92.7)	2721 (64.1)		
Total	3248	4248		

### Programmatic participation of FSW

Of the 3248 FSW recruited between 2008 and 2011, 2440 (75.1%) reported taking part in a publicly available condom distribution and education / HIV counseling & testing program, but only 974 (30.0%) of them reported attending peer education. The proportion of these two intervention services received by FSW recruited between 2012 and 2015 both increased to 91.8% and 41.9%, (*p* < .01, *p* < .01) respectively (see Table 3).

**Table 3:** The intervention service of FSW received in Period 2008–2011 and in Period 2012–2015

Variables	Period 2008–2011	Period 2012–2015	*χ* ^*2*^	*P*
Condom distribution and education / HIV counseling & testing program			3.93	< 0.01
Yes	2440 (75.1)	3900 (91.8)		
No	808 (24.9)	348 (8.2)		
Peer education			1.12	< 0.01
Yes	974 (30.0)	1780 (41.9)		
No	2274 (70.0)	2468 (58.1)		
Total	3248	4248		

### Multi-level fixed model analysis - understanding FSW and HIV risk

Preliminary analyses indicated that twelve factors were associated with differences in characteristics of FSW between the two time periods. All factors were entered into a multi-level fixed model to further understand changes in HIV risk factors over time (Period 2008–2011 = 0 or 2012–2015 = 1), level of establishment was assigned as the aggregation hierarchies. As can be seen in Table 4, older age, being married, cohabiting, and being divorced or widowed were significantly and positively associated with FSW recruited between 2012 and 2015. Answering HIV-related knowledge questions correctly, reporting consistent condom use in the last month, no illicit drug use in lifetime and no STI in the past year, and having HIV testing previously were positively related to FSW recruited in the later period. Participation in a condom distribution and education / HIV counseling & testing program and peer education programs were also related to FSW who were recruited in the later period. However, Han ethnicity and high school education level was negatively associated with being recruited in the more recent time period.

**Table 4:** Multi-level fixed model analysis for HIV infection of Period 2012–2015 compared to Period 2008–2011

Factors	*Estimate (95%CI)*	*P*
Intercept	−0.648 (−0.749, −0.547)	< 0.001
Age (continuous variable)	0.013 (0.012, 0.015)	< 0.001
Relationship status		
Single or regular sexual partner (not living together)	0	
Married	0.042 (0.015, 0.070)	0.003
Cohabiting	0.054 (0.0003, 0.108)	0.049
Divorced or widowed	0.148 (0.098, 0.199)	< 0.001
Hukou (Registered residence)		
Guangxi Province	−0.007 (− 0.013, 0.028)	0.486
Outside Guangxi	0	
Ethnicity		
Han	−0.036 (−0.069, − 0.004)	0.030
Zhuang	−0.002 (− 0.045, 0.040)	0.911
Other	0	
Education level		
Illiteracy	0	
Primary school	0.007 (−0.029, 0.044)	0.692
Middle school	0.004 (−0.035, 0.044)	0.830
High school	−0.094 (− 0.146, − 0.043)	< 0.001
College and above	− 0.069 (− 0.185, 0.047)	0.242
HIV-related knowledge		
No	0	
Yes	0.377 (0.349, 0.406)	< 0.001
Consistent condom use in the last month		
No	0	
Yes	0.173 (0.153, 0.194)	< 0.001
Lifetime illicit drug use		
Yes	0	
No	0.145 (0.072, 0.217)	< 0.001
STI diagnosis in the past year		
Yes	0	
No	0.058 (0.023, 0.093)	0.001
Lifetime HIV test		
No	0	
Yes	0.265 (0.243, 0.288)	< 0.001
Condom distribution and education / HIV counseling & testing program		
No	0	
Yes	0.072 (0.044, 0.100)	< 0.001
Peer education		
No	0	
Yes	0.088 (0.067, 0.109)	< 0.001

## Discussion

This study examined HIV prevalence among Chinese FSW living in a high-risk area of southwestern China. A secondary purpose was to examine associations between personal socio-demographics, HIV-related knowledge, HIV-related risk behaviors, and HIV. Compared to HIV prevalence among FSW worldwide, HIV prevalence in Guigang City was found to be much lower than other countries [7]. However, HIV prevalence among FSW in Guigang City was found to be higher than found in other areas of China [20]. Notably, HIV in Guigang City was found to be higher than other cities within Guangxi province [21, 22]. However, the present results indicate that year 2011 was an inflexion point for Guigang City’s HIV epidemic. This finding is similar to research conducted across Guangxi province, which found a decline of HIV among FSW across the whole province since 2011 [21]. These data from one of largest and most important cities in Guangxi support the conclusion that HIV among FSW may be decreasing [21].

The current research also adds to the literature by investigating factors related to HIV risks and how they may be changing among Chinese FSW [23, 24]. Trend analyses indicated that the HIV epidemic among FSW in Guigang City began to decrease in 2012, with population growth having little influence on HIV. [http://www.gxgg.gov.cn/] Therefore, this study compared characteristics of those recruited in the period of increasing prevalence (2008–2011) to those (2012–2015). Our results showed that age, relationship status, educational attainment, HIV-related knowledge, consistent condom use in the last month, lifetime illicit drug use, history of STI in the past year, HIV testing previously, and participation in governmental HIV prevention programs all changed between these time periods.

Differences in demographic characteristics between FSW between 2008 and 2011 and 2012–2015 included the discovery of older, and more married, cohabiting, divorced or widowed FSW. These findings are important as public health officials consider the impact of changing demographics and tier meanings for tailoring their current public health efforts. It is notable that the rise of commercial sex using internet-based and smartphone advertisements and communications were not included in this study. Internet and smartphone sue among Chinese FSW have been found to be related to demographic characteristics, condom use, and FSW use of HIV testing and counseling services [25, 26]. Future research should determine if the current sentinel surveillance venue-based recruitment efforts are sufficient to address the changing demographics of FSW in China, or if new modes of recruitment are needed.

The results also illustrate several promising and most likely inter-related factors that can be used to further understand research and programs for Chinese FSW. First, results indicate that HIV knowledge among FSW in Guigang city has improved over time. Although difficult to compare to other studies based upon differing measures, the percentage of FSW in this study who could answer six or greater questions about HIV correctly was over 90% in the 2012–2015 time period. This is encouraging as HIV knowledge has been associated with greater levels of condom use [27] and may be a sign that governmental HIV programming is having a positive impact in the FSW community. However, it must be noted that knowledge of HIV has not always been associated with interest in or improvements in HIV prevention behaviors [17, 26, 28, 29]. Second, the current study also showed higher reports of consistent condom use and greater numbers testing for HIV in the 2012–2015 time period. It is possibly that these findings are related to indications of greater use of governmental programs that distributed free condoms, provided educational materials and lessons, and peer support. Previous research has indicated that FSW in China are reporting higher rates of consistent condom use and HIV testing, [13, 30] and that these rates have both be improved through the use of behavioral interventions [13, 31]. Future studies need to more specifically examine the impact of these governmental programs by examining evaluation data that has been collected during those efforts.

There are several limitations in the present study. First, the survey was implemented each year during 2008 to 2015 and it is likely that some participants completed the study more than once. However, the venues were randomly sampled and the HIV prevalence in this study is likely to represent the HIV prevalence of the FSW population in Guigang City. Second, this study relied on self-reported survey responses. As such, underreporting of behaviors due to social stigma and discrimination and overreporting of condom use due to social desirability may have occurred.

## Conclusions

A decline in the HIV epidemic among FSW in Guigang City was observed in this study. Age, relationship status, educational attainment, HIV-related knowledge, consistent condom use in the last month, lifetime illicit drug use, history of STI in the past year, HIV testing previously, and participation in governmental HIV prevention programs were associated with this change. Results also indicate that Chinese policies on HIV/AIDS control and prevention may have a positive impact in the FSW community. Findings from this study can be used to add to these interventions by the tailoring of innovative prevention programs aimed at further reducing the disparate health outcomes among FSW. Further, it is important to expand these programs not only to cover FSW, but also consider multi-level population-based approaches including clients of FSW and their other sexual partners. Research into HIV transmission has uncovered networks of risk based upon complex individual, dyadic, familial, and social determinants. Corresponding expansions of HIV prevention and care programs may greatly improve HIV health disparities in China.

## Abbreviations

AIDS: Acquired Immune Deficiency Syndrome
AOR: Adjusted Odds Ratio
CDC: Center(s) for Disease Control and prevention
ELISA: Enzyme-Linked Immunosorbent Assay
FSW: Female Sex Workers
HIV: Human Immunodeficiency Virus
OR: Odds Ratio
SPSS: Statistical Package for the Social Science
STI: Sexually Transmitted Infections

## References

[1] Wu Z, Sullivan SG, Wang Y, Rotheram-Borus MJ, Detels R (2007). Evolution of China's response to HIV/AIDS. Lancet.

[2] Zhang L, Chow EP, Jing J, Zhuang X, Li X, He M (2013). HIV prevalence in China: integration of surveillance data and a systematic review. Lancet Infect Dis.

[3] National Health and Family Planning Commission of the People's Republic of China. 2015 China AIDS response progress report. 2015.

[4] Xiao Z, Noar SM, Zeng L (2014). Systematic review of HIV prevention interventions in China: a health communication perspective. Int J Public Health.

[5] Xiao Z, Li X, Mehrotra P (2012). HIV/sexual risk reduction interventions in China: a meta-analysis. AIDS Patient Care STDs.

[6] Shannon K, Strathdee SA, Goldenberg SM, Duff P, Mwangi P, Rusakova M (2015). Global epidemiology of HIV among female sex workers: influence of structural determinants. Lancet.

[7] Baral S, Beyrer C, Muessig K, Poteat T, Wirtz AL, Decker MR (2012). Burden of HIV among female sex workers in low-income and middle-income countries: a systematic review and meta-analysis. Lancet Infect Dis.

[8] Wu J, Li Q (2015). The profile of Guigang City. Guangxi yearbook.

[9] Wang XY, Ge XM, Tang ZZ, Shen ZY, Lan WZ, Zhu QY (2015). Epidemiological characteristics of HIV/AIDS in Guangxi during 2008–2013 [in Chinese]. J Appl Prev Med.

[10] Zhao Y, Ma Y, Chen R, Qin X, Hu Z (2015). The policy analysis of HIV/AIDS prevention and control [in Chinese]. Chinese Health Service Management.

[11] The State Council AIDS Working Committee Office, the People's Republic of China. China HIV/AIDS monitoring and evaluation framework (trial). Beijing: Beijing medical publishing house; 2007.

[12] Tucker JD, Henderson GE, Wang TF, Huang YY, Parish W, Pan SM (2005). Surplus men, sex work, and the spread of HIV in China. AIDS (London, England).

[13] Chow EP, Muessig KE, Yuan L, Wang Y, Zhang X, Zhao R (2015). Risk behaviours among female sex workers in China: a systematic review and data synthesis. PLoS One.

[14] Huang Y, Henderson GE, Pan S, Cohen MS (2004). HIV/AIDS risk among brothel-based female sex workers in China: assessing the terms, content, and knowledge of sex work. Sex Transm Dis.

[15] Zhang L, Shah IH, Li X, Zhou Y, Zhang C, Zhang L (2017). Does the use of HIV testing and counseling services influence condom use among low-paid female sex workers in Guangxi, China?. AIDS Care.

[16] Huang Z, Wang W, Martin M, Nehl E, Smith B, Wong F (2011). “Bridge population”: sex workers or their clients?–STI prevalence and risk behaviors of clients of female sex workers in China. AIDS Care.

[17] Hesketh T, Zhang J, Qiang D (2005). HIV knowledge and risk behaviour of female sex workers in Yunnan Province, China: potential as bridging groups to the general population. AIDS Care.

[18] Wang Y, Yang Y, Shi X, Mao S, Shi N, Hui X (2016). The spatial distribution pattern of human immunodeficiency virus/acquired immune deficiency syndrome in China. Geospat Health.

[19] Zhang XY, Huang T, Feng YB, Li M, Chen FF, Li YG (2015). Characteristics of the HIV/AIDS epidemic in women aged 15-49 years from 2005 to 2012 in China. Biomed Environ Sci.

[20] Zhang L, Chow EP, Su S, Yiu WL, Zhang X, Iu KI (2015). A systematic review and meta-analysis of the prevalence, trends, and geographical distribution of HIV among Chinese female sex workers (2000–2011): implications for preventing sexually transmitted HIV. Int J Infect Dis.

[21] Chen Y, Abraham Bussell S, Shen Z, Tang Z, Lan G, Zhu Q (2016). Declining inconsistent condom use but increasing HIV and syphilis prevalence among older male clients of female sex workers: analysis from sentinel surveillance sites (2010-2015), Guangxi, China. Medicine.

[22] Han L, Zhou C, Li Z, Poon AN, Rou K, Fuller S (2016). Differences in risk behaviours and HIV/STI prevalence between low-fee and medium-fee female sex workers in three provinces in China. Sex Transm Infect.

[23] Nguyen T, Stewart DE, Lee CTP, Dang TNH (2017). Prevalence of HIV infection and risk factors among female sex workers in a Southeast Province of Vietnam. Aids Behav.

[24] Porter KA, Turpin J, Begg L, Brown G, Chakhtoura N, Church E (2016). Understanding the intersection of young age, mucosal injury, and HIV susceptibility. AIDS Res Hum Retroviruses.

[25] Hong Y, Li X, Fang X, Lin X, Zhang C (2011). Internet use among female sex workers in China: implications for HIV/STI prevention. AIDS Behav.

[26] Youchun Z, Brown JD, Muessig KE, Xianxiang F, Wenzhen H (2014). Sexual health knowledge and health practices of female sex workers in Liuzhou, China, differ by size of venue. AIDS Behav.

[27] Fan YG, Liu JJ, Zhang YJ, Dai SY, Li MQ, Ye DQHIV (2015). Other sexually transmitted infections, and risk behaviors among female sex workers in Liuzhou, China. Int J Gynaecol Obstet.

[28] Chen H, Cheng F, Luan RS, Hu H, Zhang LL, Zhang JX (2003). A study on awareness of STD/AIDS risk behaviors and health care-seeking behaviors among female CSWs. J China AIDS/STD.

[29] Cai Y, Shi R, Shen T, Pei B, Jiang X, Ye X (2010). A study of HIV/AIDS related knowledge, attitude and behaviors among female sex workers in shanghai China. BMC Public Health.

[30] Muessig KE, Smith MK, Maman S, Huang Y, Chen X-S (2014). Advancing the prevention agenda for HIV and other sexually transmitted infections in South China: social science research to inform effective public health interventions. AIDS Behav.

[31] Wang B, Wang Q-Q, Yin Y-P, Liang G-J, Jiang N, Gong X-D (2012). The effect of a structural intervention for syphilis control among 3597 female sex workers: a demonstration study in South China. J Infect Dis.

